# A New Clinically Driven Classification for Acute Aortic Dissection

**DOI:** 10.3389/fsurg.2020.00037

**Published:** 2020-06-23

**Authors:** Salah D. Qanadli, Sonaz Malekzadeh, Nicolas Villard, Anne-Marie Jouannic, Daniel Bodenmann, Piergiorgio Tozzi, David C. Rotzinger

**Affiliations:** ^1^Department of Diagnostic and Interventional Radiology, Lausanne University Hospital (CHUV), Lausanne, Switzerland; ^2^Faculty of Biology and Medicine (FBM), University of Lausanne (UNIL), Lausanne, Switzerland; ^3^Department of Radiology, Kantonsspital Frauenfeld, Frauenfeld, Switzerland; ^4^Department of Heart and Vessels, Lausanne University Hospital (CHUV), Lausanne, Switzerland

**Keywords:** aortic dissection, endovascular procedures, acute disease, computed tomography angiography, selection for treatment

## Abstract

**Objectives:** To report a new classification scheme for acute aortic dissection (AAD) that considers the aortic arch as a separate entity and integrates patterns of malperfusion syndrome (MPS). The proposed classification was evaluated retrospectively in a large population.

**Materials and Methods:** We retrospectively reviewed pre-therapy CT angiograms of 226 consecutive patients (mean ± SD age: 64 ± 12 years) with AAD. AADs were reclassified with a new classification scheme that included three aortic dissection types (A, involving at least the ascending aorta; B, involving exclusively the descending aorta; and C, involving the aortic arch with/without the descending aorta) and four malperfusion grades (0: no MPS; 1: dynamic MPS; 2: static MPS; 3: static and dynamic MPS). AAD features were assessed and correlated to patient outcomes.

**Results:** According to the new classification, we identified 152 type A dissections (92 A0, 11 A1, 38 A2, 11 A3); 50 type B (38 B0, 5 B1, 6 B2, 1 B3); and 24 type C (17 C0, 6 C2, 1 C3). Type C represented 11% of all AADs. MPS occurred in 39, 24, and 29% in type A, B, and C, respectively. Type C was treated with significantly more endovascular or hybrid interventions (37%) than in types A (3%) and B (20%) (*p* < 0.001).

**Conclusion:** The new AAD classification was feasible, and type C was easily identified (“non-A, non-B”). Preliminary findings supported the usefulness of this classification for the decision-making process and subsequent treatments.

## Introduction

Acute aortic dissection (AAD) is a complex disease with a reported annual incidence of 2–15/100,000 inhabitants. AAD is associated with a high mortality rate: ~1% of patients die/h (1–4). AAD is part of the spectrum of acute aortic syndromes (AAS), defined as a tear in the intimal/media layers, which creates a new lumen (false lumen), where the blood flows between the dissecting membrane and the adventitial layer. Several well-described risk factors contribute to this condition, including advanced age, male gender, hypertension, aortic dilatation, connective tissue disorders, and bicuspid aortic valve (3, 5–8).

When left untreated, AAD can rapidly progress to a highly lethal condition, due to rupture, tamponade, myocardial infarction, or aortic valve insufficiency (9, 10). Additionally, AAD can lead

to malperfusion syndrome (MPS), which threatens the brain parenchyma, abdominal viscera, and lower limbs (11).

Different combinations of dissecting membrane extension patterns and MPS represent multiple facets of this disease, and each combination can lead to a dramatically different outcome. Previous attempts have been made to distinguish different AAD patterns by developing classifications to guide health care providers in deciding whether to apply urgent, appropriate invasive treatments or conservative alternatives. The two primary traditional classification schemes, the De Bakey and Stanford classifications, are based on the location and extent of AAD (12, 13). These classifications are simple to use and have driven patient management for a long time. However, their implementation with modern therapeutic strategies is limited because they do not include several factors that predict outcomes and might influence the decision-making process, particularly in patients with MPS. De Bakey and Stanford classifications were initially described based on surgery and transcatheter angiography; thus, they do not account for the more subtle signs detectable with cross-sectional imaging. Furthermore, by design, AADs that exclusively involve the aortic arch are not distinguished from descending AAD in the Stanford scheme, even though aortic arch AAD may require a specific management.

The objective of the present study was to establish a new classification scheme for AAD, based on computed tomography angiography (CTA). This new scheme considers the aortic arch as a separate entity, and it integrates the location and patterns of the dissecting membrane. We evaluated the feasibility and potential interest of the proposed classification in a large population of patients with AAD.

## Materials and Methods

### New Classification Principle

**Figure 1:**
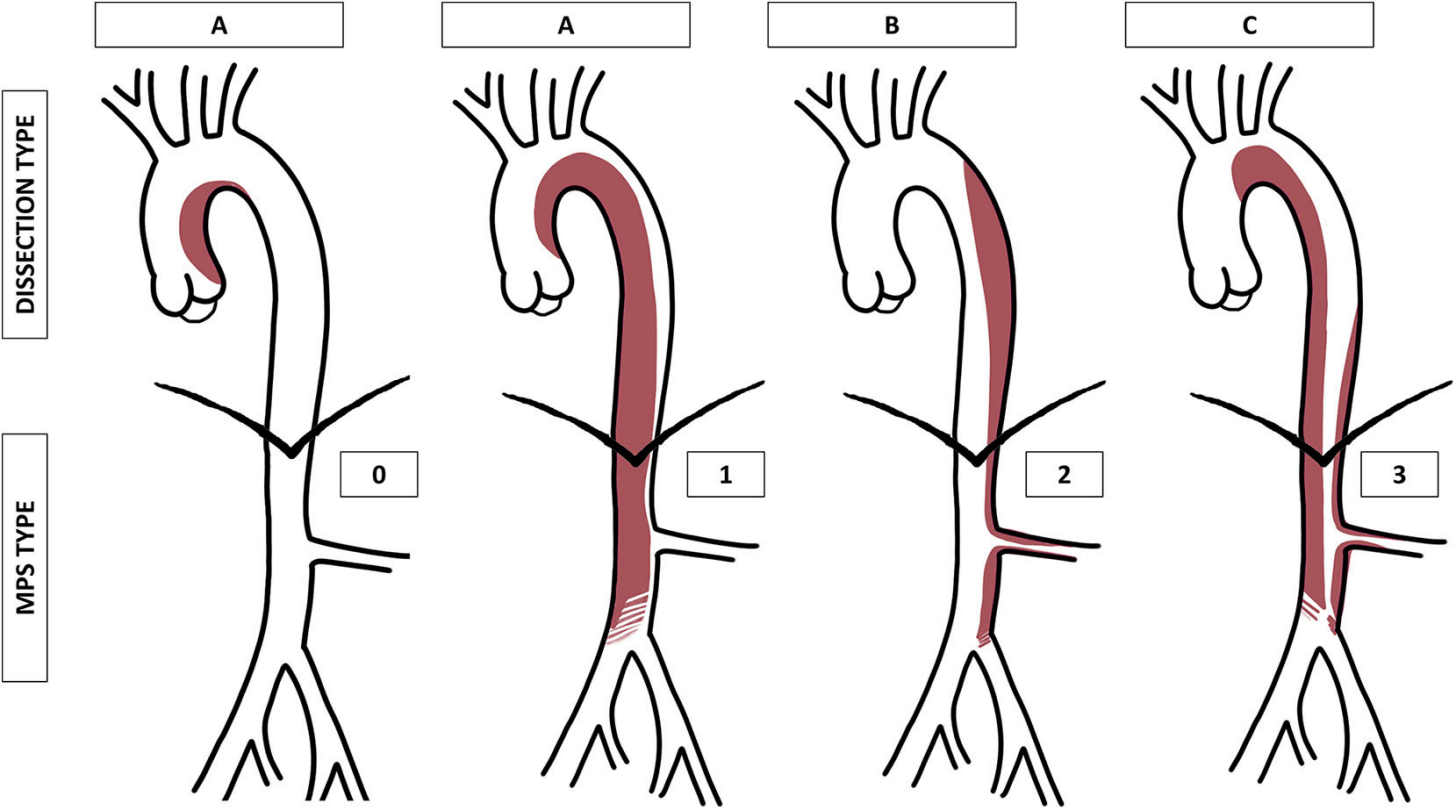
Graphical representation of the proposed aortic dissection classification (type A, B, or C) and malperfusion syndrome (MPS) subtypes (grade 0, 1, 2, or 3). MPS grade 1 is represented as a compression of the true lumen in the abdominal aorta, grade 2 as an extension of the dissection into the left renal artery, and grade 3 as a combination of both.

The proposed classification, inspired by the Stanford classification, was intended to consider the aortic segment involved in the intimal tear and dissecting membrane and the potential hemodynamic consequences of the dissection on peripheral and visceral arteries. Based on the dissecting membrane location and shape on a CTA, AADs were classified into three types and four grades (Table 1, Figure 1).

**Table 1:** Definitions of the proposed aortic dissection classification scheme.

**Types**		**Grades**	
Type A	Dissection involving at least the ascending aorta (segment I)	0	Absence of MPS
Type B	Dissection involving exclusively the descending aorta, with or without an extension to the abdominal aorta (segments III and/or IV)	1	Dynamic MPS
		2	Static MPS
Type C	Dissection involving the aortic arch with or without the descending aorta (segment II and/or III/IV)	3	Dynamic and static MPS

A dynamic MPS is an aortic dissecting membrane that covers the origins of first-order aortic branches, which hemodynamically compromises the flow, whereas a static MPS corresponds to a dissection that extends into the lumen of first-order branches and hemodynamically compromises the flow (14, 15).

### Patient Selection

This retrospective study considered all consecutive patients with *de novo* AADs identified on a CTA that were admitted to our hospital between January 2005 and December 2017. An AAD was defined as either (1) a dissection (class I AAS) that complicated or was associated with an aortic intramural hematoma (class II AAS) or (2) a penetrating atherosclerotic ulcer (class IV AAS). We excluded patients with a known history of AAD, previous aortic surgery, or an iatrogenic AAD. We also excluded patients without a pre-therapy CTA or a low-quality CTA. Our institutional review board approved the study and waived informed consent.

All medical files were reviewed for patient demographic data and cardiovascular risk factors. We collected data on all primary symptoms, including dyspnea, chest pain, focal neurological deficits, abdominal pain, and lower extremity ischemia.

### CTA Protocols and Image Analysis

Patients underwent CTA with a 64-row multidetector system (LightSpeed VCT, GE Healthcare), from January 2005 to December 2015, or with a 256-row multidetector system (Revolution CT, GE Healthcare), from January 2016 to December 2017. All CTAs followed a routine, non-ECG-gated helical mode protocol. Images were acquired in the arterial phase with the bolus tracking technique, after an intravenous administration of 100 mL iodinated contrast medium (Accupaque 300, GE Healthcare) at a flow rate of 4 mL/s, followed by a 40 mL saline flush. Patients were positioned lying down on their back, with arms placed above the head. Patients were asked to hold their breath at full inspiration during image acquisition. The acquisition parameters were: tube potential, 120 kVp; detector collimation geometry, 64 × 0.625 mm, until December 2015, and 128 × 0.625 mm, thereafter; beam pitch, 1; rotation time, 0.5 s; tube current, 400 mA; automatic exposure control, combined *xyz*-axis. The reconstruction parameters were: section thickness, 1.25 mm; section overlap, 1 mm; kernel, standard. All pre-therapy CTA examinations were reviewed by consensus between two board-certified radiologists blinded to patient symptoms and treatment allocation. Axial images and multiplanar reconstructions, available at the discretion of radiologists, were used to reclassify all AADs with the new classification.

Treatment options were stratified as conservative, endovascular, or surgical. Conservative treatment included antihypertensive therapy and medication for pain management. Endovascular procedures included stent-grafts, stents, or percutaneous aortic fenestrations. Surgical approaches referred to open surgery.

The length-of-stay (LOS) and 30-day mortality rate calculations started after the diagnosis.

### Statistical Analysis

Statistical analyses were performed with STATA statistical software (Version 13.0, October 30, 2013; StataCorp). Quantitative variables are expressed as the mean ± standard deviation, and qualitative variables are presented as raw numbers, proportions, or percentages. The Chi-square or Fisher's exact test were used to evaluate differences between categorical data as appropriate. The *t*-test or ANOVA were used to evaluate continuous data. *P* < 0.05 were considered significant.

## Results

We retrieved data for 232 consecutive patients. Six patients (2.6%) were excluded from the study, due to the absence of pre-therapy CTA or poor image quality. Consequently, 226 patients (157 men and 69 women, mean age: 64 ± 12 years, range: 24–91) were included in the study.

Patient demographic characteristics are summarized in Table 2. Patients frequently had hypertension (69%; 155/226), particularly patients with type A dissections (48%; 73/152). The most frequent primary clinical presentation was chest pain, which was observed in 62% (141/226) of patients. MPS was rarely the primary sign (<10%). Well-known aortic disorders, such as Marfan syndrome, bicuspid aortic valve, aortic stenosis, and aortic dilatation, were observed in 4, 5, 2, and 11 patients, respectively.

**Table 2:** Patient demographic data (*N* = 226).

**Characteristic**	**n (%)**
Men/women	157 (69%)/69 (31%)
**Cardiovascular risk factors**	
Hypertension	155 (68%)
Smoking	83 (37%)
Diabetes	29 (13%)
Hypercholesterolemia	41 (18%)
Prior medical history of cardiovascular disease	52 (23%)
Prior medical history of pulmonary disease	23 (10%)
**Primary clinical presentation**	
Chest pain	141 (62%)
Syncope	22 (10%)
Focal neurological deficit	20 (9%)
Dyspnea	17 (8%)
Cardiopulmonary arrest	9 (4%)
Abdominal pain	8 (3%)
No information	9 (4%)

**Figure 2:**
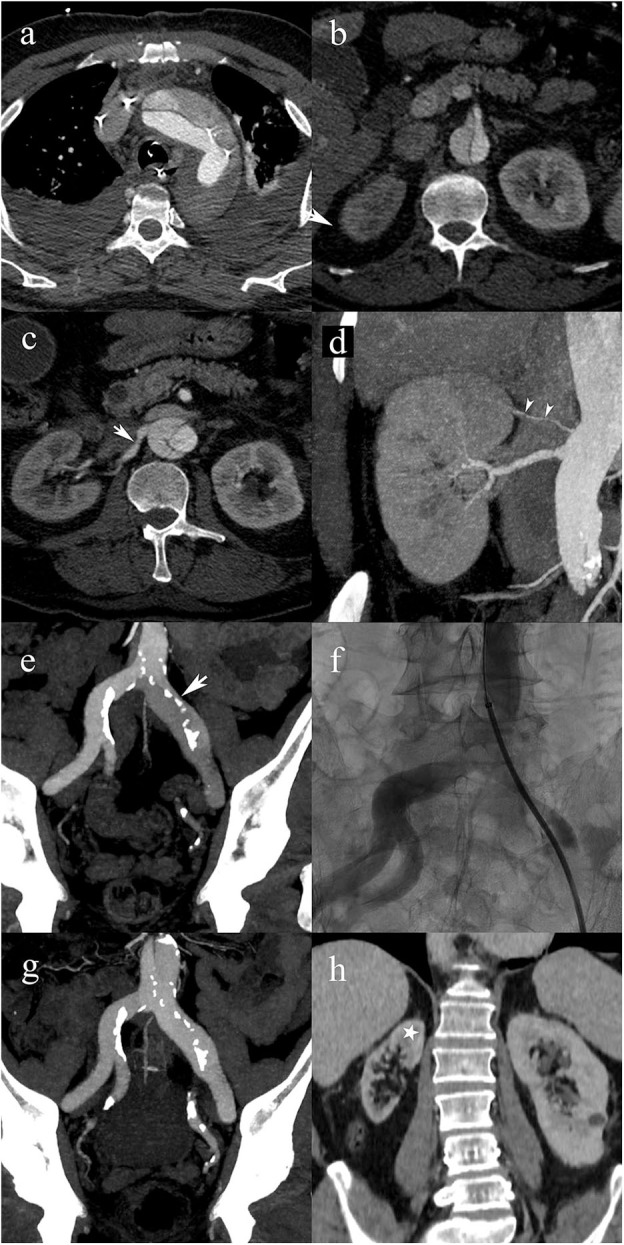
A 63-year-old male patient with type A2 AAD. Patent false lumen **(a)**, without compromise of the superior mesenteric artery **(b)**; post-ostial dissection of the right renal artery (white arrow) **(c)**; post-ostial stenosis of the right renal artery resulting in a static MPS. Note the accessory renal artery supplying the superior pole of the right kidney (white arrowheads) **(d)**. Extension into the left common iliac artery with compression of the true lumen (arrow) resulting in a static MPS of the left lower limb **(e)**; transcatheter angiography shows a significant stenosis of the true lumen in the left common iliac artery **(f)**; follow-up CTA shows that the iliac artery stenosis disappeared following endovascular fenestration procedure **(g)**; 5-year follow-up CTA shows right renal atrophy due to untreated static MPS. Cortical thinning spares the upper pole (star) because of the patent accessory renal artery.

**Figure 3:**
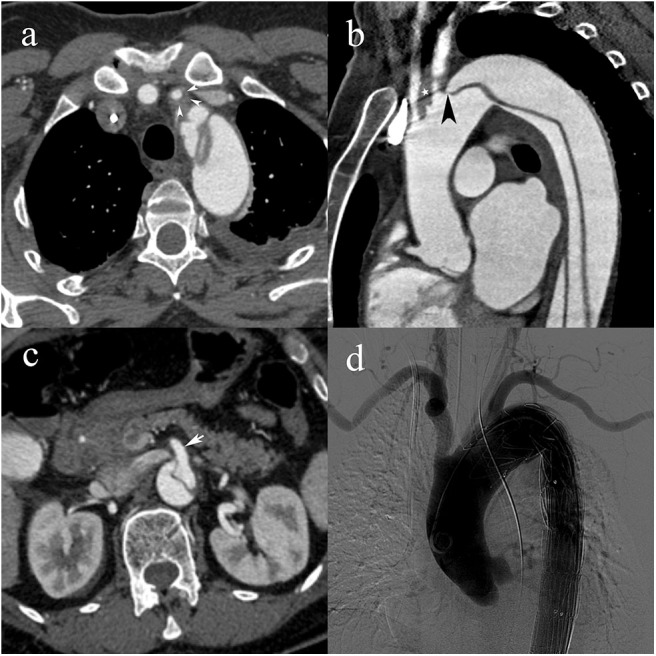
A 55-year-old woman with type C2 AAD. Dissecting membrane arising in the aortic arch with hematic infiltration causing eccentric thickening of the left common carotid artery wall (white arrowheads) **(a)**; extension to the left common carotid artery (white star) and intimomedial flap arising at the level of the left subclavian artery (black arrowhead) **(b)**; absence of vascular compromise in the superior mesenteric artery (white arrow) **(c)**; transcatheter angiography following subsequent endovascular stent-graft placement with lack of opacification of the left common carotid artery.

**Figure 4:**
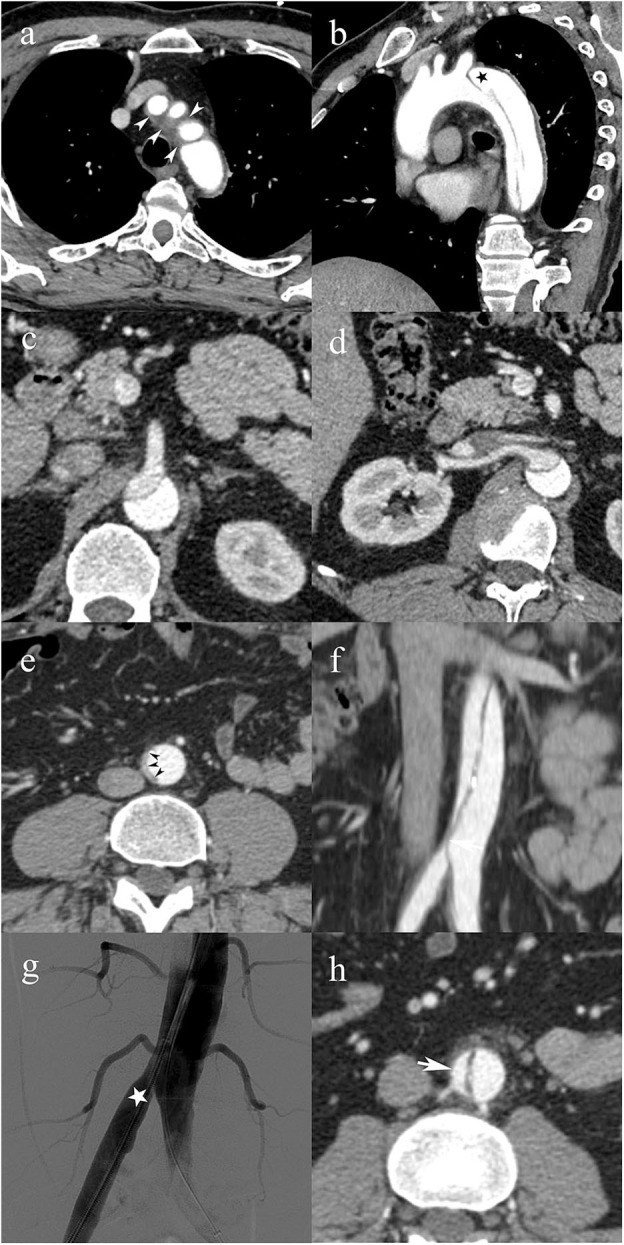
A 49-year-old male patient with type C0 AAD. Soft tissue density surrounding the origin of the supra-aortic vessels (white arrowheads) consistent with non-communicating dissection **(a)**; patent false lumen (black star) distal to the left subclavian artery **(b)**; superior mesenteric artery **(c)**, and right renal artery **(d)** arising from the false lumen with no evidence of vascular compromise; infrarenal abdominal aorta with complete collapse of the true lumen (black arrowheads) **(e)**; vascular compromise of the right common iliac artery due to the collapsed true lumen causing dynamic MPS of the right lower limb **(f)**; transcatheter angiography immediately after endovascular fenestration shows restored blood flow in the right common iliac artery **(g)**; follow-up CTA shows axial image of the infrarenal abdominal aorta with patent false lumen **(h)**.

Based on the proposed classification scheme, the most frequent dissection type was A, with a prevalence of 67% (152/226) (Table 3, Figure 2). Type C represented 11% (24/226) of patients (Figures 3, 4). Patients with type C AAD were initially classified as Stanford type A in 21% and Stanford B type in 79% (Table 4).

**Table 3:** Number of patients in each type of aortic dissection (A, B, C), and malperfusion syndrome grade.

	**New classification subtypes and management**											
	**A**				**B**				**C**			
***n*** **(%)**	152/226				50/226				24/226			
	67.30%				22.10%				10.60%			
	**A0**	**A1**	**A2**	**A3**	**B0**	**B1**	**B2**	**B3**	**C0**	**C1**	**C2**	**C3**
Conservative	4	0	1	0	35	0	2	0	7	0	2	0
Endovascular	1	0	0	0	2	3	3	0	6	0	2	1
Surgical	87	10	34	11	1	1	1	0	4	0	2	0
Hybrid	0	1	3	0	0	1	0	1	0	0	0	0
Total (%)	92/152 (60.5%)	11/152 (7.2%)	38/152 (25%)	11/152 (7.2%)	38/50 (76%)	5/50 (10%)	6/50 (12%)	1/50 (2%)	17/24 (70.8%)	0/24 (0%)	6/24 (25%)	1/24 (4.2%)

**Table 4:** Initial Stanford classifications of Type C aortic dissections and treatments (*N* = 24).

**Initial classification and treatment**	**Patients, *n* (%)**
Stanford A-Type	5 (21%)
Conservative	1
Surgery	3
Endovascular	1
Stanford B-Type	19 (79%)
Conservative	8
Surgery	3
Endovascular	8

**Figure 5:**
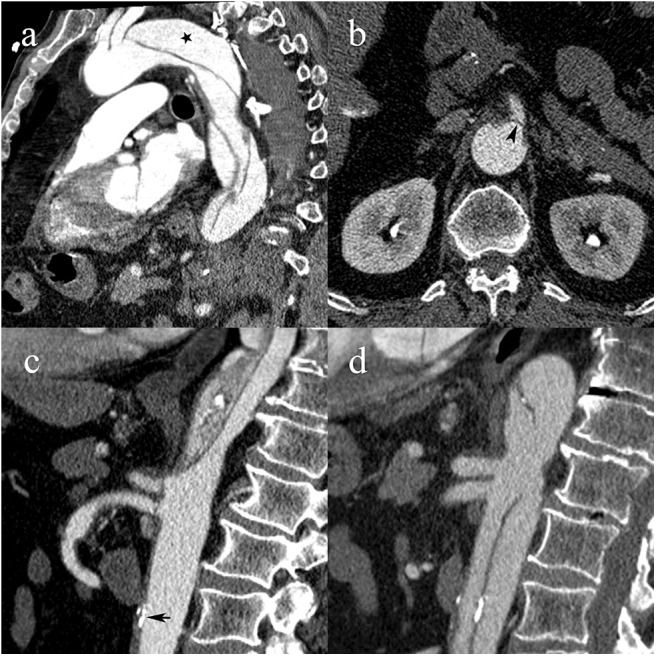
A 64-year-old male patient with type B1 AAD. Intimomedial flap arising distally to the left subclavian artery with patent false lumen (black star) **(a)**; flap prolapsing over the ostium of the superior mesenteric artery, causing dynamic MPS (black arrowhead) **(b)**; complete collapse of the true lumen due to hemodynamic forces draping the dissecting membrane against the anterior wall of the aorta and prolapsing the flap over the ostia of the celiac and superior mesenteric artery. Atherosclerotic calcifications are in line with the aortic wall (black arrow) **(c)**; follow-up CTA shows patent true lumen following urgent endovascular fenestration, restoration of celiac and superior mesenteric artery blood flow, and atherosclerotic calcifications now displaced into the lumen **(d)**.

**Figure 6:**
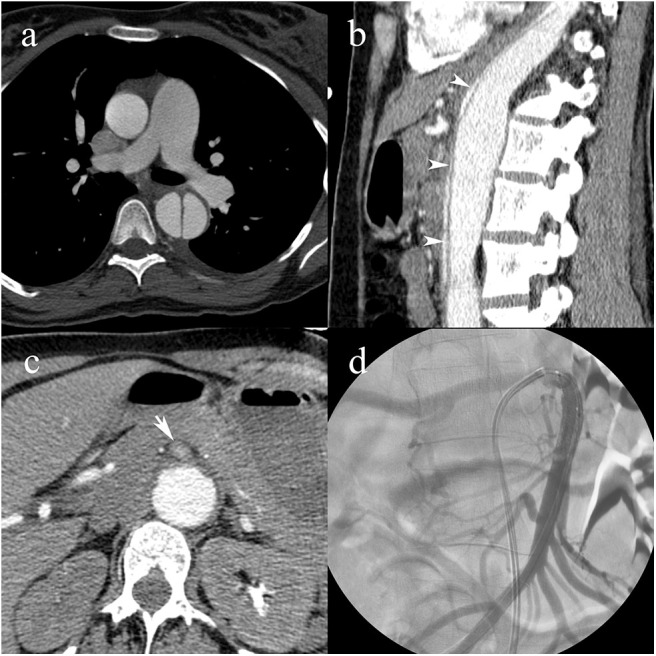
A pregnant 31-year-old female patient with a history of Marfan syndrome, having type B3 AAD. Intimomedial flap in the descending aorta **(a)**; collapse of the true lumen (white arrowheads) causing dynamic MPS of the celiac artery and extension into the superior mesenteric artery causing static MPS **(b)**; compromised blood flow in the superior mesenteric artery (white arrow) **(c)**; transcatheter angiography following angioplasty and stent placement shows patent superior mesenteric artery with blood pressures raised to systemic levels **(d)**.

An MPS was observed in 39% of patients with type A (60/152) (Table 3, Figure 2), and A2 was the most frequent MPS (38/60, 63.3%). In type B (Figures 5, 6), and C (Figure 3), the MPS rates were 24% (12/50) and 29% (7/24), respectively (Table 3). The most common MPS affected the kidney and was reported in 8% of patients (19/226).

Dissection complications, like tamponade and myocardial infarction, were reported in 7% (16/226) and 3% (8/226) of patients, respectively, and they were solely observed in type A dissections. Three patients (2%) with type A died in the emergency department after a CTA; no death was reported in patients with type B or C before treatment.

Unsurprisingly, the most common treatment for type A was open surgery (96 %, 146/152). In type B, conservative treatment was applied most frequently (74%; 37/50). In type C, endovascular treatment or surgery was the most frequent (63%; 15/24) treatment strategy. Endovascular or hybrid (endovascular and surgery) treatment was more frequently applied in type C (37%; 9/24) than in types A (3%; 5/149) and B (20%, 10/50; *p* < 0.001). The details on treatment strategies for type C AAD are summarized in Tables 3, 4.

In the subset of patients with MPS, surgical or hybrid treatment was applied in 98% (59/60) cases with type A dissection, whereas in type B 83% (10/12) patients had surgical, hybrid, or endovascular treatment and in type C 71% (5/7) had surgical, hybrid or endovascular treatment. These rates were similar for A vs. B (*p* = 0.29) and B vs. C (*p* = 0.60), but were significantly different for A vs. C (*p* = 0.03).

The mean LOSs were 17.2, 17.1, and 13.9 days in types A, B, and C, respectively, with no significant difference between groups (*p* = 0.59). In groups with and without MPS, the mean LOSs were 17.7 and 16.9 days, respectively, in type A groups; 20.4 and 16.1 days, respectively, in type B groups; and 13.3 and 14.1 days, respectively, in type C groups. The mean LOS was not significantly different among patients with MPS and patients without MPS in types A, B, and C groups (*p* = 0.74, 0.25, and 0.83, respectively). Further analysis showed no significant differences in LOS across the four MPS grades in each type of dissection (*p* = 0.94, 0.55, and 0.97 in type A, B, and C dissections, respectively).

The 30-day mortality rates were 13% (20/149), 8% (4/50), and 8% (2/24) in types A, B, and C, respectively, with no significant difference between groups (*p* = 0.50). The 30-day mortality rates in patients with and without MPS, respectively, were 17% (10/59) and 11% (10/90), in type A; 8% (1/12) and 8% (3/38), in type B; and 0 and 12% (2/17) in type C. The presence of MPS was not associated with mortality (*p* = 0.31, 0.96, and 0.34, in types A, B, and C, respectively).

## Discussion

The new classification was feasible and easy to use in a large, representative group of consecutive patients. Notably, it identified a new subgroup of patients with type C dissections that involved the aortic arch without affecting the ascending aorta.

The traditional aortic dissection classifications devised by De Bakey and Stanford are based on the intimal tear location and propagation. They mainly address the ascending and descending aorta, and they lack accuracy regarding aortic arch involvement (13). This omission has led to a dilemma concerning the treatment of aortic arch dissections, although treatments for ascending and descending aortic dissections are well-established. Arch involvement is defined as either a retrograde extension of an intimal tear in the descending aorta (which spares the ascending aorta) or an intimal tear itself located in the aortic arch.

In 1994, Von Segesser et al. (16) were the first to highlight the failure of traditional AAD classifications to take into account the aortic arch appropriately; they proposed the term “non-A non-B” to refer to aortic arch involvement in AADs. Four years later, Lansman et al. (17) suggested a modified Stanford classification by adding an aortic arch subcategory to type A and B dissections. Since then, several studies debated how to classify the retrograde extension of a descending aortic dissection; however, primary aortic arch involvement remained an open issue (18, 19). Recently, Rylski et al. (20) established a new classification that divided “non-A non-B” into descending-entry and arch-entry types. This definition was based on the location of the entry tear; the descending entry tear was located distal to the left subclavian artery; the arch entry tear was located between the innominate and left subclavian arteries. Here, we proposed a modified classification scheme that incorporated type C, defined as any arch involvement that corresponded to the “non-A non-B” type, described by Rylski et al. (20).

Previous studies reported AAD frequencies involving the aortic arch that varied between 2 and 11% of all dissections (16, 20–22) and were reported to be acute “type B” dissections in 5.4–74.2% (9, 19, 23–25). This variability in results was probably related to the lack of a standardized definition for the “non-A non-B” entity. The present study aimed to clarify the prevalence of type C AADs in a consecutive patient cohort. We found that types A, B, and C AADs had prevalences of 67, 22, and 11%, respectively. Type C was the least frequent type, inconsistent with previous reports. Among the type C AADs, 21% (5/24) were initially classified as type A, and 79% (19/24) were initially classified as type B (Table 4).

Despite its crucial clinical implications, MPS has been largely ignored in traditional classification schemes. In contrast, the classification proposed here integrated MPS by adding subcategories for dynamic and static MPS (grades of 0–3), for each type of dissection. In the present study, for each type of dissection, the most frequent MPS subtype was grade 0 (absence of MPS) and the second most frequent was grade 2 (static MPS). The most frequent result of MPS was acute renal failure. It should be noted that the MPS classification was done retrospectively, which means that MPS-related findings did not drive management and consequently, patients did not necessarily receive appropriate management. As an example, the case presented in Figure 2 shows a static MPS of the right renal artery that remained untreated with subsequent renal atrophy.

Although traditionally type A dissections are treated with surgical management, and type B dissections are treated with medical management, the recent development of endovascular methods has widely influenced these conventional approaches. Furthermore, the lack of accuracy in classifying aortic arch dissections has generated ambiguity and disagreement in treatment strategies (19, 22, 26). Several studies proposed to adopt surgical treatments for “non-A non-B” aortic dissections; those studies showed that conservative treatment was associated with a higher mortality rate (20, 21, 27). Urbanski et al. conducted a study with a predefined surgical treatment for patients with an intimal tear in the aortic arch, and conservative treatment for patients with an intimal tear in the descending aorta that extended retrogradely (but not beyond the innominate artery). Although their study was limited by the low number of patients, they demonstrated a higher mortality rate in patients that underwent conservative treatment (21). Rylski et al. (20) proposed endovascular repair for “non-A non-B” type dissections. In the present study, the most common therapeutic approach for type A dissections was surgery, and the most common approach for type B dissections was medical treatment. However, in type C dissections, 64% of patients received endovascular or surgical options. This preliminary observation highlighted the notion that type C AAD is a distinct entity that requires specific management. However, further studies are needed to confirm this statement.

Although treatments strongly depend on the type of AAD, the presence of MPS can have a substantial clinical impact, because it typically requires additional aggressive therapies. Endovascular methods for managing MPS, such as bare-metal stents, stent-graft placement, and/or intimal fenestration, provided a benefit by reducing the mortality rate (28–30). Recently, Augoustides et al. (31–33) introduced the Penn classification, which integrated localized and generalized ischemia into the traditional Stanford classification, but that classification was allocated solely to type A AAD. Our classification scheme integrated the MPS, which can change the therapeutic approach for types A, B, and C AADs. Additionally, the Penn classification remains incomplete because it disregards the MPS type (i.e., dynamic or static). Although we found no significant differences in mortality or the LOS among the different categories of AAD in the present study, we noted that patients with MPS mostly received aggressive treatment, and about two-thirds of patients with type C AAD received aggressive management (surgery or endovascular procedure).

This study had several limitations. First, the study was retrospective in nature, which could lead to an inclusion bias. The second limitation was the low number of patients, particularly in group C. The third limitation was that the population selected (patients with AAD) did not include all AASs. However, by design, our classification was also intended to be applicable to intramural hematomas and penetrating atherosclerotic ulcers. Fourth, MPS treatment could not be evaluated meaningfully because of the retrospective study design and the fact that MPS was often not reported radiologically thus not managed adequately. Fifth, the inter-observer variability was not assessed. The last limitation was related to the imaging modality used (CTA). Similar to the previous classification schemes, our classification scheme was applicable to all imaging modalities, provided that the modality could identify all the necessary criteria. More extensive studies are required to confirm the effectiveness of our new classification scheme in facilitating the decision-making process and patient management.

## Conclusion

In conclusion, we report a new classification scheme that was feasible and easy to use. The classification scheme identified “non-A non-B” dissections from the Stanford classification as type C dissections. Our preliminary findings showed that type C dissections were likely to benefit more from aggressive therapies than from medical treatment. Integrating MPS grades into the proposed classification scheme should help drive therapeutic decisions. Finally, this classification scheme took advantage of the latest developments in transcatheter therapies and could be applied to any AAS type.

## Data Availability Statement

The datasets generated for this study are available on request to the corresponding author.

## Ethics Statement

The studies involving human participants were reviewed and approved by the Ethics Committee of the Canton de Vaud. Written informed consent for participation was not required for this study in accordance with the national legislation and the institutional requirements.

## Author Contributions

All authors contributed in drafting the manuscript and revising it critically. Furthermore, they were involved in the following tasks. SQ: study design, data analysis and interpretation, and literature review. SM: analysis and interpretation, literature review. NV: data acquisition. A-MJ: data analysis. DB: data acquisition and analysis. PT: data analysis and interpretation. DR: data analysis and interpretation, literature review, and statistical analysis.

## Conflict of Interest

The authors declare that the research was conducted in the absence of any commercial or financial relationships that could be construed as a potential conflict of interest.
